# Fatigue among Long-Term Breast Cancer Survivors: A Controlled Cross-Sectional Study

**DOI:** 10.3390/cancers13061301

**Published:** 2021-03-15

**Authors:** Saskia W. M. C. Maass, Daan Brandenbarg, Liselotte M. Boerman, Peter F. M. Verhaak, Geertruida H. de Bock, Annette J. Berendsen

**Affiliations:** 1 Department of General Practice and Elderly Care Medicine, University Medical Center Groningen, University of Groningen, PO Box 196, 9700 AD Groningen, The Netherlands; d.brandenbarg@umcg.nl (D.B.); liselotte.boerman@radboudumc.nl (L.M.B.); a.j.berendsen@umcg.nl (A.J.B.); 2 NIVEL, Netherlands Institute of Health Services Research, 3513 CR Utrecht, The Netherlands; pverhaak@xs4all.nl; 3 Department of Epidemiology, University Medical Center Groningen, University of Groningen, PO Box 30001, 9700 RB Groningen, The Netherlands; g.h.de.bock@umcg.nl

**Keywords:** breast neoplasms, fatigue, cancer survivors, long-term adverse effects, anxiety disorder, depressive disorder

## Abstract

**Simple Summary:**

The number of long-term breast cancer survivors is increasing. Earlier research showed that many breast cancer survivors suffer from fatigue during and shortly after treatment. Fatigue is distressing and can severely impact quality of life. In this research, we assessed whether the prevalence of fatigue is also elevated long after breast cancer treatment. We showed that even ten years after diagnosis, one in four breast cancer survivors experience fatigue. This is more than women of the same age without a history of cancer. In addition, we found that fatigue among long-term breast cancer survivors was associated with symptoms of depression and anxiety.

**Abstract:**

Background: Fatigue is the most common and persistent symptom among women in the first five years after a breast cancer diagnosis. However, long-term prevalence of fatigue, among breast cancer survivors, needs further investigation. Aim: To compare fatigue experienced by long-term breast cancer survivors with that in a reference population and to evaluate the determinants of that fatigue. Design and Setting: A cross-sectional cohort study of 350 breast cancer survivors ≥5 years after diagnosis and a reference population of 350 women matched by age and general practitioner. Method: Fatigue was measured using the Multidimensional Fatigue Inventory (MFI-20), and a sum score of >60 (multidimensional fatigue) was the primary outcome. Logistic regression was applied to compare the prevalence of multidimensional fatigue between the survivor and reference populations, adjusted for body mass index (BMI) and for cardiovascular and psychological variables. Odds ratios (ORs) and 95% confidence intervals (95%CIs) were estimated. Logistic regression was applied to evaluate the determinants of multidimensional fatigue among the survivors. Results: Breast cancer survivors (median 10 years after diagnosis), more often experienced multidimensional fatigue than the reference population (26.6% versus 15.4%; OR, 2.0 [95%CI, 1.4–2.9]), even after adjusting for confounders. The odds of multidimensional fatigue were also higher among survivors with symptoms of depression (32.2% versus 2.7%; OR, 17.0 [95%CI, 7.1–40.5]) or anxiety (41.9% versus 10.1%; OR, 6.4 [95%CI, 3.6–11.4]). Conclusion: One in four breast cancer survivors experience multidimensional fatigue and fatigue occurs more frequently than in women of the same age and general practitioner. This fatigue appears to be associated with symptoms of depression and anxiety.

## 1. Introduction

Breast cancer is the most common cancer among women [1], and due to an ageing population, its incidence is increasing in Western countries [2]. Fortunately, better treatment regimens mean that survival has also increased over recent decades, leading to a growth in the number of long-term survivors [3]. A downside of this has been that a growing population of women are experiencing the negative long-term effects of breast cancer therapy [4]. Among these effects, fatigue is the most common complaint among survivors, with a prevalence of 40%–80% [5,6]. Fatigue can be distressing and can severely affect daily life, potentially persisting into the long term [7]. Several mechanisms have been described to explain fatigue in survivors. It may be related to the disease itself, to the treatment, or to the physical or psychological side effects [8], having known associations with anxiety, depression, and cardiac dysfunction [9,10]. Fatigue also appears to be multidimensional, with different subtypes described, including general, physical, or mental fatigue, as well as reduced activity and reduced motivation [11]. Despite being studied extensively among breast cancer survivors, little is known about the long-term prevalence of fatigue in this population. Indeed, most studies have only reported data up to 5 years after diagnosis, and they have not compared fatigue among survivors to that among women with no cancer [12,13,14,15,16]. Given that fatigue is common in the general population, and the prevalence varies between studies (10.8%–21.6%) [17,18], such a comparison is necessary to enable the accurate interpretation of the data.

In this study, we evaluated the prevalence of multidimensional fatigue, and its persistence over time, among long-term breast cancer survivors compared to a reference population. We also evaluated the association of multidimensional fatigue with factors such as breast cancer treatment, cardiac dysfunction, depression, and anxiety.

## 2. Method

### 2.1. Context

In the Netherlands, almost all inhabitants are registered with a general practitioner (GP). All GPs record clinical contacts in electronic databases in which they assign codes according to the International Classification for Primary Care, and prescribed medication is coded using the Anatomical Therapeutic Chemical classification system [19,20].

### 2.2. BLOC Study

This present study was embedded in the previously published cross-sectional BLOC study [21,22], which was designed to explore the long-term effects of breast cancer treatment on cardiac dysfunction and psychological distress. Between 2013 and 2016, we compared 350 women treated for breast cancer 5 or more years previously to 350 women with no history of cancer matched by age and GP. Women with a local/locoregional recurrence were included if the recurrence was diagnosed more than five years ago. Half of the breast cancer survivors were treated with chemotherapy (with or without radiotherapy) and the other half were treated with radiotherapy alone. After receiving written informed consent, all participants completed questionnaires, underwent echocardiography, and had their BMI calculated. Details of cancer treatment and cardiovascular diagnoses were extracted from hospital files and GP medical records. The study was registered at ClinicalTrials.gov (accessed on 25 January 2021)(ID: NCT01904331) and approved by the medical ethics committee of the University Medical Center Groningen (UMCG).

### 2.3. Current Study

For the current study, we compared multidimensional fatigue between the two cohorts and explored the factors associated with multidimensional fatigue in breast cancer survivors. These factors included age, time since breast cancer diagnosis, type of cancer treatment, systolic cardiac dysfunction, cardiovascular disease (CVD), depression, and anxiety.

#### 2.3.1. Primary Outcome

Multidimensional fatigue was measured with the Dutch version of the 20-item Multidimensional Fatigue Inventory (MFI-20) questionnaire [23]. This measures five subtypes of fatigue: general fatigue, physical fatigue, mental fatigue, reduced activity, and reduced motivation. In turn, each subtype has four questions that are each scored on a five-point Likert-scale to give subtypes scores of 4–20. Fatigue was considered present for a subtype if the subtype score was >12 [24], and multidimensional fatigue was considered present if the total score was >60 [25]. Participants were also classified as having severe multidimensional fatigue if they scored >12 on all subtypes.

#### 2.3.2. Variables

Information on CVD was obtained from the electronic medical records of the participants’ GPs. All CVD diagnoses up to the time of the cross-sectional study were included. A list of CVD codes that were included is provided in Supplementary Data S1. The presence of any of these codes in the participant’s file was taken to indicate a diagnosis. Echocardiography was also performed to measure systolic cardiac function, which we defined as a left ventricle ejection fraction (LVEF) <54%, using the biplane method of disk summation (modified Simpson’s rule) recommended by the European Association for Cardiovascular Imaging/American Society of Echocardiography (EACVI/ASE) [26].

Depression and anxiety were assessed using the Hospital Anxiety and Depression Scale (HADS), a self-reporting questionnaire that comprises subscales for symptoms of depression (HADS-D) and anxiety (HADS-A). Each subtype consists of seven items, with scores ranging from 0 to 3 per item. For this study, we used a cut-off of ≥8 per subscale to indicate the presence of depression or anxiety symptoms (includes both mild and severe). The HADS performs well in recognizing depression and anxiety disorders [27]. Contrasting with other questionnaires to identify depression or anxiety symptoms, the HADS does not include a question about tiredness or sleeping difficulties [23].

### 2.4. Statistics

Medians and interquartile ranges (IQRs) are reported to describe continuous variables, whereas numbers and percentages are reported to describe categorical variables. For the purpose of analyses, the date of breast cancer diagnosis, functioned as the index date for the matched control. Hereby, we could make a comparison between the breast cancer survivors and matched controls, including adjusting for time since diagnosis. Univariate logistic regression was used to evaluate the prevalence of multidimensional fatigue (sum score >60), and its persistence over time, among long-term survivors compared to the reference population. We set the presence of multidimensional fatigue (yes or no) as the outcome of interest and stratified the analyses according to time since diagnosis for women <10 years and those at ≥10 years after diagnosis. In multivariable logistic regression, the results were adjusted for time since diagnosis ≥10 years, BMI >30, LVEF <54%, CVD ≥1, HADS-D ≥8, and HADS-A ≥8. The presence of severe multidimensional fatigue and the fatigue subtypes were evaluated in the same way. In addition, an independent-samples *t*-test was conducted to compare the mean sum MFI score for breast cancer survivors to the mean sum MFI of the reference population. This analysis was repeated, stratified for time since diagnosis for women <10 years versus ≥10 years after breast cancer diagnosis.

We performed further univariate logistic regression analyses to evaluate the associations between being a breast cancer survivor with or without multidimensional fatigue (the outcome) and the various factors of interest (the determinants). The following factors were considered: age > 65 years at assessment, ≥10 years since diagnosis, BMI > 30, treatment (chemo- and or radiotherapy), hormonal therapy, CVD ≥ 1, LVEF < 54%, HADS-D ≥ 8, and HADS-A ≥ 8. We applied a full model in the multivariable logistic regression, adjusting for all variables and describing the absolute effects of statistically significant associations between the factors and multidimensional fatigue. This was repeated for severe multidimensional fatigue and all fatigue subtypes. All analyses were performed using IBM SPSS Statistics for Windows, Version 26.0 (IBM Corp., Armonk, NY, USA).

## 3. Results

Participants had a median age of 63 (IQR 57–68) years and breast cancer survivors had a median follow-up duration of 10 (IQR 7–14) years after diagnosis (Table 1). A total of 22 breast cancer survivors were included (≥5 years) after a local/locoregional recurrence.

### 3.1. Fatigue among Survivors and Reference Population

Breast cancer survivors experienced multidimensional fatigue significantly more often than the reference population (26.6% vs. 15.4%; OR, 2.0 [95%CI, 1.4–2.9]; Table 2). This remained for survivors <10 years after diagnosis (29.2% vs. 13.7%; OR, 2.6 [95%CI, 1.5–4.5]), but the significant difference was lost for those >10 years after diagnosis (24.3% vs. 16.9%; OR, 1.6 [95%CI, 0.95–2.6]). The mean sum score of the MFI was significantly higher for breast cancer survivors in comparison to the reference population. Moreover, breast cancer survivors in both stratified groups (time since diagnosis <10 years or ≥10 years) had significantly higher mean sum scores on the MFI than the reference population.

Severe multidimensional fatigue was significantly more prevalent among survivors than in the reference population, (6.6% vs. 1.7%; OR, 4.0 [95%CI, 1.6–10.1]), as was each fatigue subtype: general fatigue (40.7% vs. 25.4%; OR, 2.0 [95%CI, 1.5–2.8]), physical fatigue (32.6% vs. 20.9%; OR, 1.8 [95%CI, 1.3–2.6]), reduced activity (24.0% vs. 14.0%; OR, 1.9 [95%CI, 1.3–2.9]), reduced motivation (17.8% vs. 11.7%; OR, 1.6 [95%CI, 1.1–2.5]), and mental fatigue (26.3% vs. 14.3%; OR, 2.1 [95%CI, 1.5–3.1]). After adjusting for time since diagnosis (≥10 years), BMI > 30, systolic cardiac dysfunction, CVD, depression, and anxiety, the odds remained significant for all fatigue subtypes except for reduced motivation.

### 3.2. Fatigue among Breast Cancer Survivors

As shown in Table 3, symptoms of depression or anxiety were significantly more common among breast cancer survivors with (OR, 17.0 [95%CI, 7.1–40.5]) than without (OR, 6.4 [95%CI, 3.6–11.4]) multidimensional fatigue. These odds remained significant after adjusting for age, time since diagnosis (≥10 years), BMI > 30, chemo- and or radiotherapy, hormonal therapy, systolic cardiac dysfunction, CVD, depression, and anxiety. Multidimensional fatigue among breast cancer survivors was not significantly associated with cardiac dysfunction (OR, 1.3 [95%CI, 0.7–2.5]) or CVD (OR, 1.8 [95%CI, 0.97–3.3]). Finally, multidimensional fatigue was experienced by 81.1% of the survivors with symptoms of depression and by 60.0% of those with symptoms of anxiety, with both being considerably higher than the 26.6% (95%CI, 22.0–31.5%) observed among all breast cancer survivors (Table 4). Comparable data were observed for severe multidimensional fatigue and all subtypes of fatigue.

## 4. Discussion

### 4.1. Summary

A quarter of the long-term breast cancer survivors in our cohort experienced multidimensional fatigue, and this occurred significantly more often than in a reference population of the same age. After considering the time since diagnosis, survivors experienced more fatigue than the reference population. Multidimensional fatigue among survivors was significantly associated with symptoms of depression and anxiety, but not with cardiac dysfunction or CVD. Indeed, breast cancer survivors with symptoms of depression or anxiety had higher levels of all fatigue subtypes. There is a need for greater awareness of persistent multidimensional fatigue and psychological problems in these women.

### 4.2. Comparison with Existing Literature

Fatigue is known to be a persistent symptom after treatment for breast cancer [28]. In our study, we additionally confirmed that this fatigue persists for a longer period than has previously been reported. Indeed, after a median follow-up of 10 years, 26.6% of breast cancer survivors experienced multidimensional fatigue, which is consistent with previous estimates from studies with shorter follow-up periods. A meta-analysis of patients with several types of cancer, reported a pooled prevalence of fatigue of 26.9% with a maximum follow-up of 10 years [29]. Studies of breast cancer survivors ≥5 years after diagnosis report prevalence rates of 16%–35% [13,30,31,32]. These estimates are particularly noteworthy given that we included survivors with a median of 10 years’ follow-up (IQR 7–14 years), whereas other researchers have included survivors up to 7 years after diagnosis, except for Bower et al., who included patients up to 10 years after diagnosis (mean 6.3 ± 1.0 years). In addition, Cella et al. reported that, ≥5 years after diagnosis, 33% of breast cancer survivors experienced at least 2 weeks of fatigue in the month before they were interviewed [28]. The wide range of estimates can be explained by the use of different questionnaires and contrasting time periods from diagnosis.

Overall, we showed that the differences in multidimensional fatigue between breast cancer survivors and controls were statistically significant up to 10 years after diagnosis, supporting the results of studies with different methodologies [33,34]. However, in this study, also breast cancer survivors 10 or more years after diagnosis scored significantly higher on fatigue than the reference population. The relatively older age of our long-term breast cancer survivors may contribute both to their symptom burden and to the persistence of fatigue, similar to the results of a study amongst older breast cancer survivors questioned 3 years after diagnosis [35]. After 10 years of follow-up, however, any differences become less significant because of the increase in the prevalence of fatigue among the reference population. This finding was also reported in a study comparing cancer patients of different ages with the general population [36].

In the present study, symptoms of depression and anxiety were significantly associated with multidimensional fatigue among long-term breast cancer survivors. Similarly, other researchers have established an association between fatigue, depression, and anxiety in studies of breast cancer survivors over 2 years [37]. Bower et al., for example, found an association between psychological factors and adverse fatigue trajectories up to 6 years after diagnosis [38].

### 4.3. Strengths and Limitations

A strength of this study is that we performed the comparison with a reference population matched by age and GP. This approach allowed us to separate the effects of breast cancer and its treatment from the effects of ageing over the long study duration. By including a random sample of breast cancer survivors and then enquiring about fatigue, we are also able to provide a more accurate prevalence measure than had we relied on women visiting a clinic. Another strength is that we only included breast cancer survivors at least 5 years after diagnosis, which resulted in a cohort median of 10 years since diagnosis. Although previous studies have reported that fatigue is persistent, none has used such a long follow-up period. Given the increasing number of long-term breast cancer survivors, it is important to know if fatigue persists this long after diagnosis. Measuring fatigue with the MFI-20 was also beneficial because it includes multiple fatigue subtypes, allowing for these to be stratified and for key issues to be identified.

Despite the strengths of our research, it is important to note that using strict cut-offs for the fatigue and severe fatigue subtypes may have resulted in an underestimation of the true prevalence [39]. Another limitation is that GPs may have coded inconsistently, which could have led to cases of CVD being missed and an underestimation of CVD diagnoses. By matching survivors and controls by the same GP, we anticipate that the impact on the comparison should be minimized. However, absolute numbers could have been underestimated and resulted in an underpowered analysis of the association with multidimensional fatigue. In addition, we did not adjust for all known determinants for fatigue [29,40]. We also measured cardiac function by echocardiography rather than the gold standard of cardiovascular MRI [41], although this should be tempered with knowledge that measuring the ejection fraction by Simpson’s 2D echo has been shown to produce comparable results [42]. Finally, the cross-sectional design precludes any comment on the course and causality related to fatigue among breast cancer survivors.

### 4.4. Implications for Research and/or Practice

Of note, approximately a quarter of breast cancer survivors still experienced multidimensional fatigue after more than 10 years of follow-up. However, because more women in the reference population also experienced multidimensional fatigue as women aged, the statistical significance of the difference was lost over time. Future research should explore this finding and the course of fatigue in a longitudinal study.

Regarding the clinical implications, our findings indicate that GPs should be vigilant for long-term (multidimensional) fatigue. Other research has shown that physical activity and exercise improve cancer-related fatigue, depression, and overall quality of life [43]. If fatigue is related to depression or anxiety, these could be modifiable factors that could improve fatigue if targeted appropriately. Fortunately, treatment options for depression and anxiety have proven to be as effective in cancer survivors as in the general population [44]; for example, cognitive behavioral therapy has been proven effective in breast cancer survivors [45]. It was also evident that depression and anxiety were associated with reduced motivation among breast cancer survivors, possibly implying that survivors with fatigue and reduced motivation may have depression or anxiety, or that stimulating motivation may be a necessary first step towards improvement.

## Figures and Tables

**Table 1:** Characteristics of breast cancer survivors and a reference population at the time of assessment [21,22].

	Breast Cancer Survivors ***** (*n* = 350)	Controls (*n* = 350)
Age; years, median (IQR)	63 (57–68)	63 (57–68)
Time since diagnosis of breast cancer or index date; years, median (IQR)	10 (7–14)	10 (7–14)
BMI > 30, *n* (%)	69 (19.7%)	70 (20%)
	*n* (%)	*n* (%)
Breast cancer stage		
Stage 0	13 (3.7%)	
Stage I	123 (35.1%)	
Stage II	161 (46%)	
Stage III	28 (8%)	
Unknown	25 (7.1%)	
Breast cancer treatment *		
Chemotherapy ***	175 (50.0%)	–
Radiotherapy ****	295 (84.3%)	–
Hormonal therapy	146 (41.7%)	–
Variables		
LVEF < 54%	52 (15.3%)	24 (7.0%)
CVD ≥ 1	54 (15.4%)	31 (8.9%)
Cardiac risk factor **	139 (39.7%)	135 (38.6%)
HADS-D ≥ 8	37 (10.6%)	17 (4.9%)
HADS-A ≥ 8	65 (18.6%)	57 (16.3%)

* as registered in the hospital and general practitioners’ files. IQR = interquartile range; BMI = body mass index; LVEF = left ventricle ejection fraction; CVD = cardiovascular diseases; HADS = Hospital Anxiety and Depression Scale. ** Cardiac risk factors: diabetes, hypertension, or dyslipidemia. *** Including breast cancer survivors who have received chemo- and radiotherapy *n* = 120 (68.6%), and breast cancer survivors who have received chemotherapy and hormonal therapy *n* = 109 (62.3%). **** Including breast cancer survivors who have received radiotherapy and hormonal therapy, *n* = 37 (21.1%). ***** Including 22 breast cancer survivors ≥ 5 years after a local/locoregional recurrence.

**Table 2:** Scores on fatigue amongst breast cancer survivors (*n* = 350) in comparison to a reference population (*n* = 350), measured with the MFI-20 questionnaire.

	Breast Cancer Survivors *n* = 350	Reference Population *n* = 350	Univariate Analyses OR (95%CI) *	Multivariable Analyses ** OR (95%CI)
**Multidimensional fatigue**				
All women (*n* = 350) Sum score >60, *n* (%) Sum score, mean (SD) ****	93 (26.6%) **49.0 (17.9)**	54 (15.4%) **42.7 (15.7)**	**2.0 (1.4–2.9)**	**1.7 (1.1–2.6**)
Time since diagnosis < 10 yrs (*n* = 161) Sum score, mean (SD) Age: median 61 (IQR 55–67) years FU: median 7 (IQR 6–8) years	47 (29.2%) **50.9 (19.1)**	22 (13.7%) **41.3 (14.6)**	**2.6 (1.5–4.5)**	**2.0 (1.0–3.7)**
Time since diagnosis ≥ 10 yrs (*n* = 189) Sum score, mean (SD) Age: median 65 (IQR 58–69) years FU: median 14 (IQR 11–17) years	46 (24.3%) **47.3 (16.8)**	32 (16.9%) **43.8 (16.5)**	1.6 (0.95–2.6)	1.5 (0.8–2.7)
**Severe multidimensional fatigue**				
All 5 subtypes scores >12, *n* (%)	23 (6.6%)	6 (1.7%)	**4.0 (1.6–10.1)**	**3.1 (1.2–8.3)**
**Fatigue, subtypes**				
General fatigue Median (25–75%) *n* (% >12) ***	11 (7–15) 142 (40.7%)	9 (5–13) 89 (25.4%)	**2.0 (1.5–2.8)**	**1.9 (1.4–2.8)**
Physical fatigue Median (25–75%) *n* (% >12)	10 (6–14) 114 (32.6%)	10 (6–14) 73 (20.9%)	**1.8 (1.3–2.6)**	**1.6 (1.1–2.3)**
Reduced activity Median (25–75%) *n* (% >12)	9 (6–12) 84 (24.0%)	9 (6–12) 49 (14.0%)	**1.9 (1.3–2.9)**	**1.7 (1.1–2.6)**
Reduced motivation Median (25–75%) *n* (% >12)	8 (5–11.5) 62 (17.8%)	8 (5–11.5) 41 (11.7%)	**1.6 (1.1–2.5)**	1.3 (0.8–2.1)
Mental fatigue Median (25–75%) *n* (% >12)	8 (5–13) 92 (26.3%)	8 (5–13) 50 (14.3%)	**2.1 (1.5–3.1)**	**2.0 (1.3–3.1)**

* Significant results are in bold. MFI = Multidimensional Fatigue Inventory; IQR = interquartile range; FU = follow-up; BMI = body mass index; LVEF = left ventricle ejection fraction; CVD = cardiovascular diseases; HADS = Hospital Anxiety and Depression Scale; OR = odds ratio; CI = confidence intervals. ** Multivariable analysis for breast cancer adjusted for time since diagnosis ≥10 years, BMI > 30, LVEF > 54%, CVD ≥ 1, HADS-D ≥ 8, HADS-A ≥ 8. *** Definitions: fatigue, subtype score >12; multidimensional fatigue, overall score >60; severe multidimensional fatigue, all subtypes score >12. **** Tested with the independent-samples *t*-test, significant *p* < 0.05.

**Table 3:** Comparison of breast cancer survivors with (*n* = 93) and without (*n* = 257) multidimensional fatigue **.

		Survivors with Multidimensional Fatigue *n* = 93	Survivors without Multidimensional Fatigue *n* = 257	Univariate Analyses	Multivariable Analyses ^a^
		***n* (%)**	***n* (%)**	OR (95%CI) *	**OR (95%CI)**
Age at time of assessment >65 years	No Yes	54 (58.1%) 39 (41.9%)	162 (63.0%) 95 (37.0%)	1 1.2 (0.8–2.0)	1 1.2 (0.6–2.2)
Time since diagnosis breast cancer ≥10 years	No Yes	47 (50.5%) 46 (49.5%)	114 (44.4%) 143 (55.6%)	1 0.8 (0.5–1.3)	1 0.8 (0.5–1.5)
BMI > 30	No Yes	80 (86.0%) 13 (14.0%)	201 (78.2%) 56 (21.8%)	1 0.6 (0.3–1.1)	1 0.5 (0.2–1.2)
CT (+/−RT)	No Yes	44 (47.3%) 49 (52.7%)	131 (51.0%) 126 (49.0%)	1 1.2 (0.7–1.9)	1 1.0 (0.6–2.0)
Hormonal therapy	No Yes	47 (50.5%) 46 (49.5%)	152 (59.1%) 105 (40.9%)	1 1.4 (0.9–2.3)	1 1.3 (0.7–2.4)
LVEF < 54%	No Yes	72 (77.4%) 16 (17.2%)	215 (83.7%) 36 (14.0%)	1 1.3 (0.7–2.5)	1 0.9 (0.4–1.9)
CVD ≥ 1	No Yes	73 (78.5%) 18 (19.4%)	223 (86.8%) 34 (13.2%)	1 1.8 (0.97–3.3)	1 1.9 (0.9–4.0)
HADS-Depression ≥ 8	No Yes	63 (67.7%) 30 (32.3%)	250 (97.3%) 7 (2.7%)	**1** **17.0 (7.1–40.5)**	**1** **8.9 (3.4–23.5)**
HADS-Anxiety ≥ 8	No Yes	54 (58.1%) 39 (41.9%)	231 (89.9%) 26 (10.1%)	**1** **6.4 (3.6–11.4)**	**1** **3.7 (1.9–7.3)**

* Significant results are in bold. **^a^** Adjusted for: age at the time of assessment > 65 years, time since diagnosis ≥10 years, BMI > 30, treatment (chemo- and or radiotherapy), hormonal therapy, LVEF > 54%, a diagnosis of CVD, HADS-D ≥ 8, HADS-A ≥ 8. BMI = body mass index; RT = radiotherapy; CT = chemotherapy; LVEF = left ventricle ejection fraction; CVD = cardiovascular diseases; HADS = Hospital Anxiety and Depression Scale; OR = odds ratio; CI = confidence intervals. ** Definition: multidimensional fatigue, overall score >60.

**Table 4:** Fatigue among breast cancer survivors (*n* = 350) overall and stratified for women with symptoms of depression (*n* = 37) or anxiety (*n* = 65).

	All Breast Cancer Survivors *n* = 350	HADS-Depression ≥ 8 *n* = 37	HADS-Anxiety ≥ 8 *n* = 65
	*n* (%; 95%CI’s) *	*n* (%)	*n* (%)
Multidimensional fatigue	93 (26.6%; 22.0–31.5)	**30 (81.1%)**	**39 (60.0%)**
Severe multidimensional fatigue	23 (6.6%; 4.2–9.7)	**10 (27.8%)**	**13 (20.3%)**
Fatigue, subtypes			
General fatigue	142 (40.7%; 35.4–45.9%)	**30 (83.3%)**	**45 (70.3%)**
Physical fatigue	114 (32.6%; 27.7–37.8)	**28 (75.7%)**	**39 (60.0%)**
Mental fatigue	92 (26.3%; 21.7–31.2)	**27 (73.0%)**	**44 (67.7%)**
Reduced activity	84 (24.0%; 19.6–28.8)	**22 (59.5%)**	**28 (43.1%)**
Reduced motivation	62 (17.8%; 13.9–22.1)	**23 (62.2%)**	**26 (40.0%)**

HADS = Hospital Anxiety and Depression Scale; CI = confidence intervals. * Bold if the percentage with fatigue is higher than the upper bound of the 95%CI for the group ‘All breast cancer survivors’.

## Data Availability

The anonymized data presented in this study are available on request from the corresponding author.

## Supplementary Materials

The following are available online at https://www.mdpi.com/2072-6694/13/6/1301/s1, Supplementary Data S1. International Classification of Primary Care Codes for Cardiovascular Diseases.
