# Supplementary material for: Fatigue among Long-Term Breast Cancer Survivors: A Controlled Cross-Sectional Study
Source: Cancers (Basel). 2021 Mar 15;13(6):1301. doi: 10.3390/cancers13061301 (PMC8001130; doi:10.3390/cancers13061301)
Supplement: Supplementary file 1 [file cancers-13-01301-s001.pdf]

cancers

Supplementary materials: Fatigue among Long-Term Breast Cancer Survivors: A Controlled Cross-Sectional Study

Saskia W. M. C. Maass, Daan Brandenbarg, Liselotte M. Boerman, Peter F. M. Verhaak, Geertruida H. de Bock and Annette J. Berendsen

Supplementary Data 1. International Classification of Primary Care Codes for Cardiovascular Diseases: Stable and unstable angina pectoris (K74), acute myocardial infarction (K75), other chronic ischemic diseases (K76), heart failure (K77), atrial fibrillation (K78), paroxysmal tachycardia (K79), non-rheumatic valve dysfunction (K83), other heart diseases (Wolff–Parkinson–White syndrome, atrioventricular block, cardiomyopathy, long QT-syndrome) (K84), transient ischemic attack (K89), cerebrovascular accident (K90) and coronary sclerosis (K91).
